# Persistence of a sugar-rejecting cockroach genotype under various dietary regimes

**DOI:** 10.1038/srep46361

**Published:** 2017-04-13

**Authors:** Kim Jensen, Ayako Wada-Katsumata, Coby Schal, Jules Silverman

**Affiliations:** 1 Department of Entomology and Plant Pathology, North Carolina State University, Raleigh, NC 27695-7613, USA; 2 W.M. Keck Center for Behavioral Biology, North Carolina State University, Raleigh, NC 27695-7613, USA

## Abstract

Glucose-aversion is a heritable trait that evolved in a number of German cockroach (*Blattella germanica* L.) populations in response to strong selection with glucose-containing insecticide baits. However, in the absence of glucose-containing bait, glucose-averse (GA) cockroaches have lower performance than wild-type (WT) cockroaches in several fitness-determining traits. We allocated 48 caged populations initiated with homozygous GA and WT adults to four dietary treatments consisting of either pure rodent chow, rodent chow mixed to yield a content of either 20% glucose or 20% fructose, or a treatment consisting of choice between the 20% glucose- and the 20% fructose-containing food. After 6 months we found significantly higher frequency of WT individuals in populations restricted to the 20% glucose food, and after 12 months all dietary treatments contained significantly more WT individuals than expected. In accompanying experiments, we found lower survival and longer development time of GA nymphs restricted to glucose-containing food. We furthermore found evidence for assortative mating of females with males from their own genotype, with significant differences within WT cockroaches. Our study shows experimental evidence that within heterogeneous populations, WT German cockroaches will over time prevail in abundance over GA individuals, even when glucose is not a dietary component.

Populations adapt to their local environments, and this contributes to the maintenance of genetic polymorphism by contemporary adaptations as specific conditions often differ between habitats and over time^1^,^2^,^3^,^4^. Adaptations that rapidly increase fitness within a population may be caused by a gain in alleles that serve a certain function, but may also be caused by the loss of such alleles and their function^5^. For instance, adaptations to environmental hazards are often associated with fitness-related costs^6^,^7^,^8^,^9^,^10^,^11^, and the genetic structure of populations may respond rapidly not only to the presence but also to the absence of particular hazards^12^,^13^,^14^. An example of rapid evolution in response to environmental hazards is the dynamics of insecticide resistance alleles within insect pest populations^15^. However, the evolution of physiological resistance to toxic constituents often incurs costs to fitness^6^,^7^,^8^,^9^,^10^,^16^,^17^, and the genetic structure of populations may thus respond rapidly to the presence of toxin by an increase in the frequency of resistance alleles, but may also respond rapidly to the absence of toxin by the loss of resistance alleles if these incur a cost to fitness^12^,^13^,^14^. In contrast, it has been little investigated whether the evolution of a behavioural aversion with no associated physiological resistance mechanism involves fitness costs, and adaptive population dynamics caused by an evolutionary change in taste reception genes following toxin removal have to our knowledge not been documented experimentally.

The evolution of dietary breadth is based on the chemophysiology of taste perception, which is genetically determined and forms the basis for dietary specialization^18^,^19^,^20^. Behavioural genetics underlying reductions or expansions in dietary breadth are thus regulated by the selective forces that favour either aversion or stimulation by certain food components that may be either detrimental or beneficial if ingested^21^. In insects, the taste system has evolved to be highly sensitive to compounds that are often toxic or associated with toxins^22^, and a strong aversion to such compounds prevents the ingestion of deleterious or lethal toxins. It has been little investigated, however, whether the evolution of taste aversions is associated with fitness-related costs and whether aversion-causing alleles decline in populations when the toxin is not present as a dietary constituent.

The German cockroach (*Blattella germanica* L.) is a universal pest in human-built structures^23^, occupying a patchily distributed habitat with efficient dispersal barriers between local populations^24^,^25^. Many tactics have been used to control populations of this insect, and the application of insecticides in bait formulations has proven highly efficacious for targeted control^26^. However, many populations have independently evolved physiological resistance mechanisms against the insecticides in baits and some have evolved behavioural mechanisms as well^27^,^28^,^29^,^30^,^31^,^32^,^33^,^34^,^35^. In response to persistent anthropogenic selection with glucose-containing baits, populations of the German cockroach have evolved a strong aversion to glucose^28^,^30^. This novel adaptation prevents consumption of toxins in glucose-containing bait, and functions by a change in the response of taste neurons on the chemosensory appendages^36^; glucose stimulates bitter receptor neurons in glucose-averse (GA) cockroaches, and the signals from these neurons override those from the sweet receptor neurons^37^. Foods containing glucose above a very low detection limit are therefore rejected by GA cockroaches, thus protecting them from ingesting the insecticide contained in the bait^28^,^30^. The adaptation is semi-dominant and autosomal, i.e. controlled by a single allele in a single major gene, and even 48 h starved homozygous and heterozygous GA cockroaches reject high concentrations of glucose^28^,^38^. However, if no other food is available over a long time, both GA genotypes will ingest glucose in small amounts^39^. Ingestion (or injection) of glucose has no toxic effects in GA cockroaches^36^.

Although glucose aversion is highly advantageous in the presence of glucose-containing baits, it appears that there are costs associated with this adaptation. GA nymphs had substantially lower survival and longer development time than wild-type (WT) nymphs when developing on a glucose-containing diet^40^,^41^. Development time was also significantly longer in GA nymphs that developed on a standard laboratory diet with glucose content below their detection limit^40^, although this was not the case for nymphs developing on artificial glucose-free diets^41^. In addition, we showed that newly eclosed adult GA females matured their oöcytes at a slower rate than WT females on the same laboratory diet, delaying the attainment of sexual receptivity^42^. Furthermore, in two recent studies we found that GA males had significantly smaller body mass than WT males developing on the same standard laboratory diet^43^,^44^. Smaller body mass is often a disadvantage in sexual selection due to lower performance under male-male competition and female choice^45^. Overall, several studies indicate lower fitness of GA than WT cockroaches in the absence of insecticide, which predicts a relative increase in WT frequency over time as an evolutionary response even in the absence of glucose.

We conducted experimental cage studies to assess the evolutionary population dynamics between GA and WT German cockroaches over one year on different dietary treatments. We hypothesized that the frequency of WT relative to GA individuals would increase over time to proportions higher than predicted under Hardy-Weinberg equilibrium (25%), and that this increase would be faster when glucose was an important dietary constituent. We furthermore compared nymphal survival and development time as well as male mating success and female fecundity between homozygous GA and WT cockroaches to explore possible mechanisms that underlie the evolutionary responses in the experimental populations.

## Materials and Methods

### Cockroaches

The GA and WT German cockroaches used in the experiment were both originally collected in Florida, USA. The GA cockroaches (T164) were collected in Gainesville in 1989^28^, and the WT cockroaches (Orlando Normal) were collected in Orlando more than 60 years ago and have since been maintained as a standard, unselected laboratory culture^28^. Cultures of both were maintained in the laboratory on *ad libitum* water and rodent chow (Purina 5001 Rodent Diet; PMI Nutrition International, St. Louis, MO, USA) under conditions similar to those described by Jensen *et al*.^42^, including monthly selection of the GA population by adding glucose-containing insecticide (hydramethylnon) bait to the containers for 2 days. This selection regime is sufficient to prevent the establishment of WT individuals from accidental introduction and maintain the frequency of glucose aversion coding alleles at near 100%. Both cultures were distributed over 4 transparent plastic containers (46 cm × 23 cm × 30 cm) for 6 months prior to experiments and maintained without selection with bait. Experiments were conducted in a room at 25–30 °C.

### Genotyping glucose-averse (GA) and wild-type (WT) individuals

Prior to experiments, 100% homozygosity of the GA and WT allele was confirmed by assaying 25 adult males from each culture container. The males from each culture container were maintained in a transparent plastic container (18 cm × 12 cm × 8 cm) with a six-piece egg carton lid for harborage and provided for two days with *ad libitum* rodent chow and water in a glass tube (2.5 cm diameter × 14.0 cm length), plugged with cotton. The food and water were then removed, depriving the cockroaches of food or water for another two days. The male cockroaches were then given two hours of a choice between a blue-dyed (erioglaucine, Sigma-Aldrich) glucose solution in 1% agar and red-dyed (Allura red, Sigma-Aldrich) water in 1% agar^27^. WT males were given a choice of 3 M glucose solution vs. water, and GA males were given a choice of 0.3 M glucose solution vs. water, based on diagnostic concentrations that result in acceptance and rejection by the two genotypes, respectively^28^,^36^. Subsequent to feeding it was confirmed that all WT males had blue guts and all GA males had red guts. Since the phenotype for glucose-aversion is determined entirely by the genotype, phenotypic assessment of glucose aversion in German cockroaches also reveals the genotype: 3 M glucose vs. water separates WT and GA cockroaches, and 0.3–0.5 M glucose vs. water separates heterozygous and homozygous GA cockroaches, both with high confidence^28^,^36^,^37^.

### Experimental foods

We produced three foods based on ground and sieved rodent chow, which contains 23.9% protein, 5% fat, 31.9% starch, 3.70% sucrose, 2.01% lactose, and very low levels of glucose (0.22% = 12.2 mM) and fructose (0.30% = 16.6 mM)^41^. All foods contained 4% agar (Table 1). One of the foods consisted only of rodent chow and agar, while the other two foods contained 20% (1.1 M) glucose or 20% (1.1 M) fructose by mass (Table 1). Since glucose and fructose are pure carbohydrates, the nutritional protein to carbohydrate (P:C) balance of these foods was adjusted to the same ratio as in rodent chow (P:C = 1:1.6^41^) by adding pure protein (Table 1). A 2:1:1 ratio of casein:peptone:albumin was used as this provides a good balance of different proteins^42^,^46^. To compensate for the micronutrient dilution incurred by adding pure macronutrients, we also added cholesterol, salts and vitamins in ratios relative to the added carbohydrate and protein that are used as optimal ratios in artificial diets^42^,^46^ (Table 1). These additions most likely differ from the values of the corresponding micronutrients in rodent chow, but were included to compensate for their potential shortage.

### Experimental populations and dietary treatments

A total of 48 populations were set up, each receiving one of four dietary treatments (*n* = 12 populations per dietary treatment). Three of the diets consisted of only one of the prepared foods, i.e. either rodent chow in agar, glucose-containing food, or fructose-containing food (Table 1), while the cockroaches in the fourth dietary treatment were given a choice between the glucose-containing food and the fructose-containing food until one or both foods were eaten. Each population was initiated with 5 adult virgin females and 5 adult virgin males from each of the two colonies that had all emerged as adults within the preceding 4 days. Although this is a relatively small founder population and genetic drift therefore might have increased variation between populations, the likelihood for directional drift across populations within dietary treatments was small because of the relatively high number of populations. Food and water were replaced every 7 days. Food was provided in restricted amounts of approximately 16 g dry food per feeding, equaling the content of one Petri dish (8.5 cm diameter × 1.5 cm height) before drying. The amount was chosen to initially provide *ad libitum* feeding conditions but also to induce competition for food once populations had substantially expanded. In the choice treatment, the 16 g of total food was divided into 8 g of the glucose-containing food and 8 g of the fructose-containing food. One or two replicates were set up for each treatment on the same day, and the complete experiment was set up over a period of two months. Prior to experiments, nymphs from the GA and WT colonies were collected into 4 transparent plastic containers (18 cm × 12 cm × 8 cm) for each colony to ensure that the progenies from multiple females were randomly mixed. Populations were kept in white, circular plastic buckets (25 cm diameter × 35 cm height) containing 6 six-piece egg cartons for harborage. A fine mesh was inserted in the cage lids to facilitate air exchange. Water was provided *ad libitum* in two cotton-plugged glass tubes (2.5 cm diameter × 14.0 cm length).

### Assaying population structure and size

The frequencies of the WT and GA genotypes were assessed at 6, 9, and 12 months after the start of the experiment. At each of the three assessments, all adult males in the population were collected into a transparent plastic container (18 cm × 12 cm × 8 cm) containing a six-piece egg carton lid for harborage and provided for two days with *ad libitum* rodent chow and water in a glass tube (2.5 cm diameter × 14.0 cm length), plugged with cotton. The food and water were then removed, depriving the cockroaches of food and water for two days. The male cockroaches were then given two hours to choose between a blue-dyed 3 M glucose solution with 1% agar and water only with 1% agar. Both homozygous and heterozygous GA cockroaches are deterred from ingesting 3 M glucose, whereas homozygous WT cockroaches ingest it in large quantities, dyeing their gut blue^36^. To ensure that cockroaches with undyed guts did not refrain from eating because they were sick, they were offered blue-dyed 3 M fructose with 1% agar, and those consuming the solution were assessed as GA while the remainder (<10%) were discarded from the assessment. The proportion of WT cockroaches in the population was then calculated as the number of males with blue gut in the first assay divided by the total number with blue gut in both assays. Only adult males were assayed because the methods used for genetic assessment of adult males are well established and verified^28^,^36^,^37^. A similar method for genotyping adult females has furthermore not been developed and verified and might be sensitive to the reproductive stage of the females, which causes very different nutritional requirements and feeding behaviours between females at different reproductive stages. Moreover, assaying males only gives a good estimate of the adult population composition, and the adult females and nymphs were required to continue the populations. Although we did not have to kill the male cockroaches to assay their gut colour, assayed males were not returned to the populations since the assay could affect fitness unevenly depending on genotype due to genotype-specific consumption differences under the assay. Not returning the adult males also allowed a level of outflow from the populations, which reduced competition for food and most likely facilitated higher nymphal survival. It also ensured that coming sires within the populations originated from the latest generation, which promoted a faster evolutionary response. At the end of the 12 month experiment, adult males were removed for assaying, the rest of the populations were killed by freezing at −18 °C, and the number of nymphs, adult females, and adult males within each population was counted.

### Effects of genotype and diet on juvenile survival and development time to the adult stage

To test whether selection for one genotype over another was likely to happen at the nymphal stage, we measured survival and development time of WT and GA nymphs from the source cultures on each of the three experimental foods (totaling six genotype and diet combinations). Females carrying oöthecae were collected from across the source culture containers and kept in two transparent plastic containers (18 cm × 12 cm × 8 cm) for each genotype (WT and GA). Nymphs from each genotype (*n* = 360) were collected within four days of hatching and distributed at random across 18 plastic containers per genotype (*n* = 20 nymphs per container). Water was present *ad libitum* in a glass tube (2.5 cm diameter × 14.0 cm length), plugged with cotton, and the lid of a six-piece egg carton was provided for harborage. The nymphs were then provided with one of the three foods *ad libitum* throughout development (*n* = 6 containers per genotype and diet). Foods were continuously resupplied to ensure constant availability. Newly eclosed adult cockroaches were recorded within 24 h of emergence and collected until all surviving nymphs had eclosed.

### Siring bias

To test whether a bias in male mating success might affect the genetic composition of offspring, we assayed the offspring phenotype of homozygous females given access to a homozygous male of each genotype. Last instar nymphs from the WT and GA genotypes were collected from across the respective cultures, kept separately in two transparent plastic containers (18 cm × 12 cm × 8 cm) for each genotype, and emerging adult females (*n* = 80 per genotype) were collected within 24 h of eclosion and transferred to individual transparent glass jars (10 cm diameter × 10 cm height). The jars contained *ad libitum* rodent chow and water provided in a cotton-plugged glass tube (9 mm diameter × 75 mm length). A piece of egg carton was added for harborage. The jars were covered with paper towel squares held in place with rubber bands. The inner walls of the jars were lined with a thin layer of petroleum jelly and mineral oil mixture to prevent climbing. At seven days of age, one WT male and one GA male taken from across the four respective culture containers were introduced overnight to each female and removed the following morning. Male cockroaches deposit a spermatophore in the female’s bursa copulatrix which prevents a second male from mating the female until the spermatophore is dropped after about 12 hours^47^,^48^. This is enough time to prevent females from re-mating within our experimental procedure, and we could therefore be confident that the offspring produced by a female were sired by only one of the two males. Each female had *ad libitum* access to rodent chow and water until her offspring hatched, and the offspring were allowed to grow to the third instar. They were then deprived of food and water for 24 h and allowed to feed for two hours from two available foods: a blue-dyed glucose solution with 1% agar in distilled water and red-dyed 1% agar in water. Whereas we assayed the main populations to distinguish WT (homozygote) vs. GA (homozygote plus heretozygote) genotypes, offspring from the homozygous GA females had to be assayed using a lower glucose concentration to distinguish homozygous and heterozygous GA nymphs. Offspring from WT females (either all homozygous WT or all heterozygotes) were thus given a choice of 3 M glucose solution vs. water to distinguish homozygous WT and heterozygous offspring, and offspring from GA females (either all homozygous GA or all heterozygotes) were given a choice of 0.5 M glucose solution vs. water to distinguish heterozygous and homozygous GA offspring, based on accepted and rejected concentrations of the respective genotypes^28^,^36^. The nymphs were assayed by determining the colour of their gut through a transparent section of the cuticle.

### Female fecundity

To test whether homozygous WT and GA females were equally fecund, five oötheca-carrying females were collected from each of the four WT and GA source culture containers (*n* = 20 per genotype) and maintained individually in transparent plastic containers (18 cm × 12 cm × 8 cm) with *ad libitum* rodent chow and a six-piece egg carton lid for harborage. Water was provided *ad libitum* in a cotton-plugged glass tube (2.5 cm diameter × 14.0 cm length). The number of offspring per oötheca was counted within 24 h after hatching.

### Statistical analyses

The numbers of individuals in the populations were compared among dietary treatments using analysis of variance (ANOVA). The proportion of WT individuals in the populations was used in the analysis of population composition (% WT vs. GA) using proportional hazard tests after arcsine transformation^49^, and all dietary groups and time points were compared using a Wilcoxon test followed by Wilcoxon comparison of each pair. Within each diet and time point, the recorded proportion of WT individuals was compared to an expected proportion of 25% (the proportion of WT homozygotes under Hardy-Weinberg expectations), using a Wilcoxon Signed-Rank test on untransformed data, based on the null hypothesis that WT and GA individuals were equally fit. The effects of genotype and diet combinations on survival were compared using a Wilcoxon test followed by Wilcoxon comparison of each pair. Effects of individual genotype, diet, and sex on development time, followed by effects of genotype and diet within each sex, were analyzed using proportional hazard tests, and all groups of individual genotype, diet, and sex were compared using a Wilcoxon test followed by Wilcoxon comparison of each pair. Proportional hazard and Wilcoxon tests were used because these data were generally not normally distributed (Shapiro-Wilk test, *p* < 0.05). Female mating bias was analyzed using likelihood ratio tests. The number of offspring hatching per oötheca was compared between genotypes using a Wilcoxon test because these data were likewise not normally distributed. The significance level was set at *α* = 0.05 in all tests. All statistical analyses were performed in JMP 13.0.0 (SAS Institute Inc., Cary, NC, USA).

## Results

### Population metrics

By the end of the experiment, 12 months after each population started with 5 adult males and 5 adult females from each of the WT and GA colonies (i.e., 20 cockroaches), the populations on average contained 2,156 ± 101 (mean ± SE) individuals with no significant difference among the four dietary treatments (ANOVA: *F*_3,48_ = 0.4535, *p* = 0.7161). Out of these, 1,721 ± 83 individuals were nymphs, 299 ± 17 were adult females, and 135 ± 13 were adult males. Note however that all adult males had been removed at 6 and 9 months. There were no significant differences in the number of individuals within life stage or sex among the dietary treatments (all *p* > 0.15).

### Population structure

**Figure 1:**
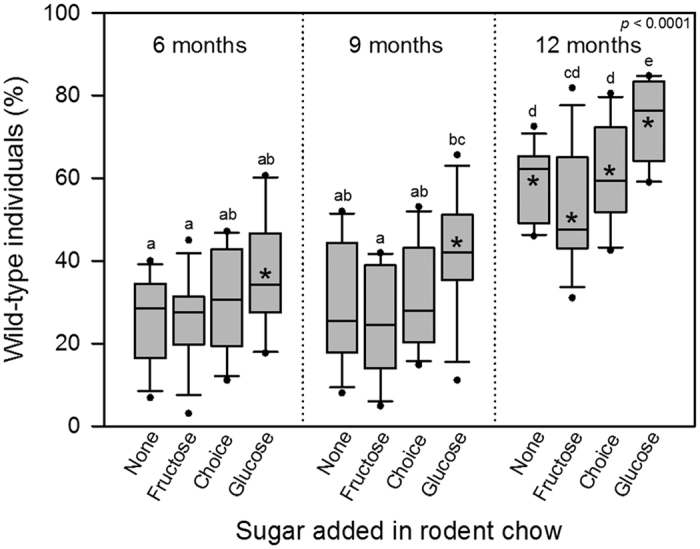
Percentage of wild-type relative to glucose-averse individuals after 6, 9, and 12 months in experimental populations. Boxes show median and 10^th^, 25^th^, 75^th^ and 90^th^ percentiles plus outliers. The choice diet consisted of a half/half combination of fructose-supplemented and glucose-supplemented rodent chow. Diets were provided in a total amount of 16 g each 7 days. Populations were initiated with five adult females and five adult males from each genotype, all within four days of eclosion. The overall *p*-value is from a Wilcoxon test. Different letters indicate significant differences (Wilcoxon pairwise comparisons, α = 0.05). Asterisks indicate a significantly higher proportion of wild-type individuals than expected (25%) if the genotypes were equally fit (Wilcoxon Signed-Rank test, α = 0.05).

Population composition was significantly affected by both diet and time, but not their interaction (Table 2, Fig. 1). After 6 and 9 months, populations given diets consisting of rodent chow, fructose-containing rodent chow, or a choice of fructose- and glucose-containing rodent chow did not deviate from population compositions expected if WT and GA individuals were equally fit (Fig. 1). However, at all three sampling intervals populations restricted to glucose-containing food consisted of significantly more WT individuals than expected from the null hypothesis (Fig. 1). At 12 months, populations consisted of significantly more WT than GA individuals within all dietary treatments (Fig. 1), still with significantly higher proportion of WT relative to GA individuals in populations restricted to glucose-containing food (Fig. 1).

### Survival to the adult stage

**Figure 2:**
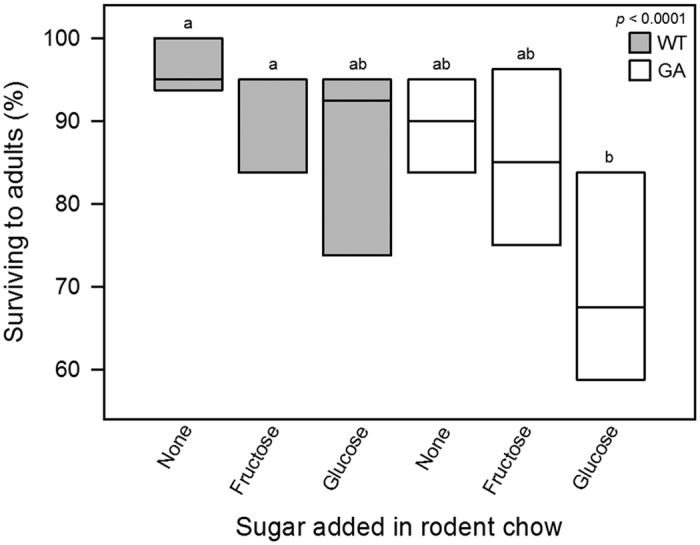
Survival of nymphs to the adult stage. Boxes show median and 25^th^ and 75^th^ percentiles including 10^th^ and 90^th^ percentiles and all individual points. Nymphs were set up in groups of 20 within four days of hatching. The overall *p*-value is from a Wilcoxon test. Different letters indicate significant differences (Wilcoxon pairwise comparisons, α = 0.05). WT, wild-type cockroaches; GA, glucose-averse cockroaches.

Analysis across genotype and diet combinations showed significant differences in survival to the adult stage (Fig. 2). Survival was lower in GA nymphs than in WT nymphs within all diets and significantly lower in GA nymphs restricted to glucose-containing rodent chow than in WT nymphs given rodent chow or fructose-containing rodent chow (Fig. 2).

### Development time

**Figure 3:**
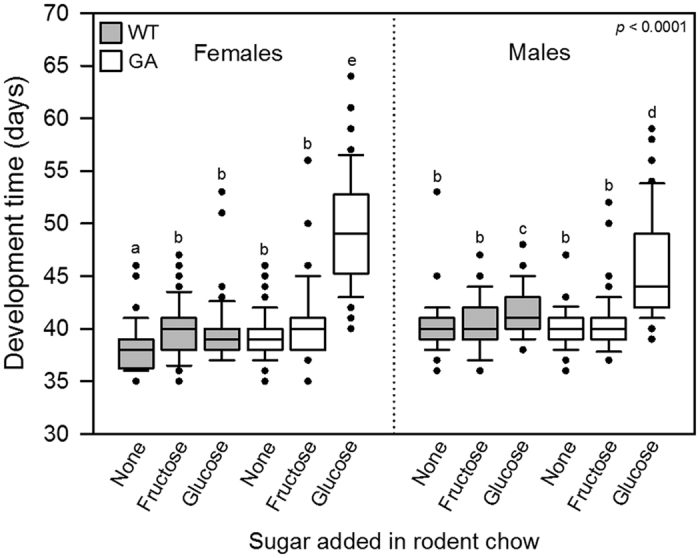
Development time of nymphs from setup as first instars to the adult stage. Boxes show median and 10^th^, 25^th^, 75^th^ and 90^th^ percentiles plus outliers. Nymphs were set up in groups of 20 within four days of hatching. The overall *p*-value is from a Wilcoxon test. Different letters indicate significant differences (Wilcoxon pairwise comparisons, α = 0.05). WT, wild-type cockroaches; GA, glucose-averse cockroaches.

Development time to the adult stage was significantly affected by genotype, diet, sex, and their individual interactions (Table 3), with more pronounced effect of genotype in females than in males (Table 4). GA nymphs restricted to glucose-containing food spent significantly longer time in development than WT nymphs and GA nymphs provided with rodent chow or fructose-containing rodent chow (Fig. 3). Female GA nymphs spent longer time in development than male GA nymphs when restricted to glucose-containing food (Fig. 3). Female WT nymphs provided with rodent chow developed significantly faster than any of the other nymphs (Fig. 3). Interestingly, male WT nymphs provided with glucose-containing food had significantly longer development time than all other nymphs except GA nymphs restricted to glucose-containing food (Fig. 3).

### Sire bias

**Figure 4:**
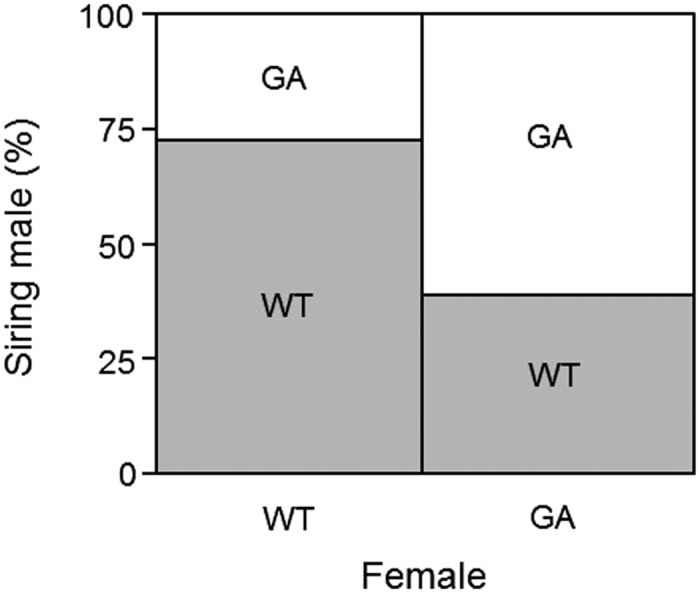
Percentage of wild-type (WT) and glucose-averse (GA) males siring offspring by WT and GA females. One WT and one GA male were introduced simultaneously to each individual female at the female age of seven days post-eclosion and left overnight, and the resulting offspring were assayed for glucose aversion to identify the siring male.

Out of the 80 WT females and 80 GA females, 64 females (80%) from each had produced offspring by day 50. WT and GA males did not sire offspring at random (Likelihood ratio test: *χ*^2^_1,128_ = 14.2273, *p* = 0.0002). Females from both genotypes produced offspring more frequently with males from their own genotype (Fig. 4), with significant bias in WT females (*χ*^2^_1,64_ = 11.39, *p* = 0.0007) but not in GA females (*χ*^2^_1,64_ = 3.01, *p* = 0.0826).

### Female fecundity

The number of offspring hatching per female oötheca (median = 40) did not differ significantly between WT and GA females (Wilcoxon: *χ*^2^_1,40_ = 0.8771, *p* = 0.3490).

## Discussion

The genotypic structure of local populations may change rapidly in response to environmental conditions as populations respond to the presence or absence of hazards in their environment^1^,^6^,^12^,^13^,^14^,^15^,^16^,^17^,^50^. In German cockroach populations, glucose aversion has evolved as an adaptation that facilitates survival in the presence of glucose-containing insecticide baits by preventing bait ingestion^28^, but the adaptation appears to be associated with a number of costs that would be expected to lower fitness of GA cockroaches^40^,^41^,^42^. We tested the relative fitness of the GA and WT genotypes within the same population in the absence of bait but on a restricted amount of food. The proportion of GA cockroaches declined significantly after 9 months within all dietary treatments, and significantly sooner when glucose was an unavoidable diet component. GA cockroaches therefore appear to be less fit and the frequency of this trait declines in heterogeneous populations under competitive conditions in the absence of bait. Our accompanying experiments showed lower survival and longer development time by GA nymphs when developing on glucose-containing rodent chow but not on rodent chow alone or fructose-containing chow. Interestingly, we found evidence of assortative mating with significantly higher siring rate of WT males than GA males in WT females, which might in part explain the relative increase in WT individuals within the populations.

Our finding that the GA genotype decreased in frequency within populations over time relative to the WT genotype regardless of diet supports earlier reports that GA cockroaches are less fit than WT individuals^40^,^42^. The decline in the frequency of GA individuals was fastest when populations were provided only glucose-containing rodent chow, which makes good sense since GA cockroaches would consume little of this food^40^,^42^, and their fitness would therefore be most severely compromised. Both the present and an earlier study^40^ showed that nymphal survival and development time were negatively affected in GA nymphs restricted to glucose-containing diets. In addition, GA females do not mature their oöcytes when restricted to glucose-containing food^42^, and GA males restricted to glucose-containing diets have slower sexual maturation evidenced by later onset of courtship behaviour^43^. It is therefore not surprising that a decline in the proportion of GA individuals, reflecting lower fitness of this genotype, could be measured at our earliest assessment after six months in populations restricted to glucose-containing food. Whereas the evolutionary response was rapid within populations restricted to glucose-containing food, it came much later when glucose-free foods were provided (Fig. 1), which was related to lower selection pressure against GA cockroaches in the absence of dietary glucose. This was even the case in the choice treatment where the fructose-containing food was most likely consumed much sooner than the glucose-containing food. The limited amount of glucose-free food, and continued access to glucose-containing food once the glucose-free food was consumed, was therefore apparently enough to sustain GA cockroaches at similar levels as in populations provided only glucose-free food.

An earlier study reported longer development time and slower oöcyte maturation in GA than in WT cockroaches even in the absence of dietary glucose^40^. In the present experiment however, we did not find slower development in GA nymphs provided with glucose-free food, yet we found rapid changes in genotype composition between 9 and 12 months also in glucose-free dietary treatments. It is likely that density gradually increased in the populations, increasingly limiting food availability and intensifying competition. Under competitive circumstances, selection is stronger since individuals must struggle to gain resources. Lower consumption capacity by GA individuals while food is still available therefore could affect overall consumption, for example, in addition to fighting for space around the food. In this situation, already well-fed individuals have a competitive advantage and less competitive individuals may be excluded from feeding, which facilitates rapid evolution. Observations in the populations after 9 months revealed that primarily adult females were able to gain access to the food. The food was furthermore quickly consumed at this stage, within approximately two days.

An additional explanation for the increasing predominance of the WT genotype over time is suggested from our experiment on potential mating bias, which indicated significantly higher mating frequency of WT females with WT males. Although both genotypes of females produced offspring more frequently with males from their own genotype, the assortative mating effect appeared stronger for WT females. Differences in body size between males from the two genotypes might cause the skew in preference strength, as WT males have larger body mass than GA males^43^,^44^, and larger males in various species typically perform better under female choice and in male-male competition for access to mates^45^. Alternatively, differences could be explained by differential success upon mating due to sire-specific abortion of the oötheca, or the two genotypes could be partially incompatible upon mating due to endosymbiont incompatibility^51^. The WT genotype might therefore in part increase over time because WT males are more successful at mating due to female choice, male-male competition, and/or incompatibility. In contrast, we did not find differences in the number of offspring produced per oötheca between females from the two genotypes, indicating that selection for the WT genotype over time is not caused by different fecundity of WT and GA females.

Finally, it is possible that slower sexual maturation in female GA cockroaches might have affected population structure over time, particularly when females were restricted to feed on glucose-containing food^42^. In addition, delayed sexual maturation in male GA cockroaches that only had access to glucose-containing food^43^ may have influenced mating success of these males and given WT males a mating advantage from the start of the experiment. On glucose-free diets, GA and WT males reach sexual maturity at the same age^43^, and differences in male sexual maturation are therefore not likely to explain the changes in population structure. However, if WT females mated selectively with WT males, it is possible that some GA females mated with already mated WT males, received little sperm, and therefore aborted their first oötheca which would delay the production of offspring by this genotype as females would require about 15 days until they were sexually receptive again^48^.

WT males had longer development time on glucose-containing than on glucose-free food (Fig. 3), and there was a tendency for lower survival on glucose-containing than on glucose-free food in WT nymphs (Fig. 2), suggesting that glucose is a less optimal dietary energy source also in WT cockroaches. A study in the moth *Manduca sexta* L. found that larvae developing on glucose-containing diets had slower development than larvae developing on sucrose- or fructose-containing diets^52^, supporting the possibility that glucose is less optimal than other energy sources when ingested at a high ratio. It has also been proposed that cockroaches and other decomposers may be sensitive to high glucose levels as this may indicate the presence of toxic cyanogenic glucosides produced by decomposing bacteria^41^. Since glucose aversion appears to have evolved independently in multiple American, European, and Asian populations of the German cockroach^28^,^30^,^37^,^53^, it is possible that the allele that codes for glucose aversion is present at low frequency in many populations and might be of ancestral origin related to interactions with microbes or plants^41^.

In addition to their greater difficulty in finding food and in attaining a balanced diet because they reject glucose^54^, GA cockroaches also appear less physiologically fit and less able to optimally exploit their environment, at least under conditions where they are under high pressure to gain resources before these are consumed and exploited by WT individuals. However, the GA genotype can be sustained in the population if food is limited overall but some glucose-free food is available. Overall, our study indicates that over a longer term, without selection with glucose-containing insecticide baits, glucose-accepting cockroaches would dominate the structure of the population, and glucose-rejecting cockroaches would be present only at very low frequency. It would be interesting to conduct population level experiments addressing the minimal frequency of intermittent treatment with glucose-containing insecticide bait that would give GA individuals an adaptive advantage. Secondly, although the present experiment shows a decline in the frequency of GA individuals including heterozygotes, it would be interesting to further investigate the costs and benefits of heterozygosity for the glucose-averse trait under variable conditions relative to homozygous individuals. It would also be interesting to test whether earlier or more severe food limitation than in the present experiment would cause faster prevalence of the WT genotype.

In conclusion, this study shows that GA German cockroaches are less fit than WT cockroaches under competitive conditions, also in the absence of dietary glucose, and that in the absence of insecticide the WT genotype will dominate over time within populations. This is to our knowledge the first demonstration that the loss of taste aversion alleles to widen dietary breadth may increase population fitness even in the absence of the aversion-causing dietary constituent. Our findings have implications for understanding the evolution of the behavioural components that underpin toxin resistance, i.e. avoidance^55^, and the pattern of a loss of aversion alleles when no toxin is coupled with the aversion-causing constituent parallels the pattern of loss of physiological resistance alleles in insect pest populations when insecticide treatment is halted^12^,^13^,^14^,^16^. This is successfully implemented in agricultural integrated pest management by ensuring the presence of pesticide free refuges in time and space, which prevent the fixation of resistance alleles within pest populations^16^,^17^,^56^,^57^. Our study suggests that a similar refuge approach may be feasible to prevent the fixation of glucose aversion alleles in German cockroach populations, and to insecticide aversion alleles that prevent the ingestion of toxin in other insect pests.

## Additional Information

**How to cite this article:** Jensen, K. *et al*. Persistence of a sugar-rejecting cockroach genotype under various dietary regimes. *Sci. Rep.* **7**, 46361; doi: 10.1038/srep46361 (2017).

**Publisher's note:** Springer Nature remains neutral with regard to jurisdictional claims in published maps and institutional affiliations.

## Figures and Tables

**Table 1:** Ingredient compositions (g/kg) of the three experimental foods.

Ingredients	Rodent chow	Fructose food	Glucose food
Rodent chow	960.0	670.0	670.0
Glucose	0.0	0.0	200.0
Fructose	0.0	200.0	0.0
Protein*	0.0	80.0	80.0
Casein	0.0	40.0	40.0
Peptone	0.0	20.0	20.0
Albumin	0.0	20.0	20.0
Cholesterol	0.0	1.7	1.7
Salts^†^	0.0	7.0	7.0
Vitamins^‡^	0.0	1.3	1.3
Agar	40.0	40.0	40.0

*Includes casein, peptone, and albumin. ^†^Wesson’s salt mixture. ^‡^Vanderzant modification vitamin mixture.

**Table 2:** Proportional hazard tests on the effects of diet and time on the composition of WT and GA individuals in the experimental populations.

Factor		*df*	*χ*^2^	*p*
Diet	Overall	3	34.4648	<0.0001
Time	Overall	2	112.4713	<0.0001
Diet × time	Overall	6	2.1077	0.9095
				
	Time			
Diet	6 months	3	8.1696	0.0426
	9 months	3	8.8603	0.0312
	12 months	3	13.1112	0.0044
				
	Diet			
Time	Rodent chow	2	27.5165	<0.0001
	Fructose food	2	25.8043	<0.0001
	Choice combination	2	27.7781	<0.0001
	Glucose food	2	29.6567	<0.0001

All diets were provided in total amounts of 16 g each 7 days. The choice combination diet consisted of 8 g fructose food and 8 g glucose food each 7 days.

**Table 3:** Proportional hazard test on the effects of genotype, diet, and sex on development time to the adult stage.

Factor	*df*	*χ*^2^	*p*
Genotype	1	41.1584	<0.0001
Diet	2	107.0222	<0.0001
Sex	1	5.2880	0.0215
Genotype × diet	2	38.6475	<0.0001
Genotype × sex	1	12.2816	0.0005
Diet × sex	2	7.6254	0.0221
Genotype × diet × sex	2	2.6628	0.2641

**Table 4:** Proportional hazard tests on the effects of genotype and diet on development time to the adult stage within each sex.

Factor	Females		*p*	Males		*p*
	*df*	*χ*^2^		*df*	*χ*^2^	
Genotype	1	40.9156	<0.0001	1	4.4660	0.0346
Diet	2	62.1698	<0.0001	2	47.0674	<0.0001
Genotype × diet	2	26.7219	<0.0001	2	11.7119	0.0029
